# Iman Nuwayhid: on building and rebuilding public health in Beirut

**DOI:** 10.2471/BLT.21.030621

**Published:** 2021-06-01

## Abstract

Iman Nuwayhid talks to Gary Humphreys about building a cross-disciplinary public health institution out of the rubble of war and efforts to rebuild Beirut in the aftermath of the August 2020 explosion.

*Q: You pursued a public health education right after medical school. What prompted that move?*

A: I was politically vocal as an undergraduate, which got me into trouble at universities in Lebanon and then in Iraq, actually getting me expelled from the university the second time. I found it hard not to question and challenge everything surrounding medicine and health and that was not always appreciated. At the advice of one of my mentors, I decided to cross the bridge from medicine to public health which was a much better fit for me because it allowed me to consider the broader determinants of health, the broader context. I chose occupational health as a focus.

*Q: You did your master’s and doctorate at Johns Hopkins but returned to Lebanon in 1991 when the country was emerging from the civil war. Was that an easy decision?*

A: For me, yes although I was married with two little children. I enjoyed my time in the USA and could have continued there, but it was important for me to come back and be a part of my country’s reconstruction and rehabilitation after 15 years of civil war. It was a shock though. The health and health-care system needs were enormous. I stuck to my specialization and remember people laughing at me when I gave my first talks about occupational health and safety. They had just come out of a war in which 150 000 people had been killed and I was talking about avoiding work injuries! So, there was a period of adjustment. But I found a home in AUB (the American University of Beirut) where I joined the Faculty of Health Sciences (FHS). I was only 35 at the time but I was almost immediately appointed to senior advisory positions. It was a great opportunity, but sad also; the senior faculty members had all retired or left the country during the war. There were so few mentors and nobody with occupational health experience. 

*Q: Emerging from that difficult period FHS has come to be recognized as one of the leading public health institutes in the Eastern Mediterranean Region. How do you account for its success?*

A: Well, first you need to remember that FHS dates back to 1954 and had built a reputation prior to and even during the civil war. But it’s true we did have to build it again, not just getting FHS back on its feet, but defining our role and our relationships with government agencies, and other disciplines and institutions. From my perspective, the turning point for FHS was the appointment of Dr Huda Zurayk as dean in 1998. She was a highly acclaimed public health academic and a leading reproductive health researcher who successfully conducted community-based research about women in Egypt, working with a wide range of people from other disciplines, including sociologists, economists and even activists. As dean, she promoted interdisciplinary exchange and encouraged the different departments to work together. Under her leadership, the faculty shifted from a traditional disease-oriented, public health institution to something more focused on population groups, e.g., older adults, youth, workers, migrants and women. That allowed us to look at the political and socioeconomic determinants of health, addressing issues of justice, equity and rights. Of course, the value of this approach has since been widely accepted and adopted by many institutions, including the World Health Organization (WHO), but it was quite new back then. I had the honour of working closely with Dr Zurayk and when I took over from her in 2008, I carried that work forward restructuring the institute to implement what we call a 360° systems-network approach.

“It is not enough to rebuild; we need to build better, build fairer.”

*Q: Can you say more about that?*

A: Essentially, we take a holistic, cross-disciplinary impact-oriented approach to public health that is articulated at the intersection of the three main areas of activity, namely research, practice and policy, with teaching at the centre. The term network is used to capture the collaborative nature of our activities. We network with others, and by others I mean sister institutions, including, I am proud to say, Birzeit University in the occupied Palestinian territory, nongovernmental organizations, United Nations agencies, government agencies and communities. 

*Q: Where do you feel FHS has had the greatest impact?*

A: Probably by encouraging regional and South-South collaborations. As a research institute we have played an important role in bringing researchers from different countries and regions together, breaking down barriers between disciplines and institutions. The FHS Center for Research on Population and Health has become the hub for several regional research networks and working groups addressing reproductive health, childbirth, tobacco control and agricultural health. We have also led efforts to sharpen the research focus on the region, notably with our work on *Public Health in the Arab World*. By the time that book went to press there were 80-plus authors involved and, to encourage their continued collaboration, we established the PHAW (Public Health in the Arab World) list which now has 2000 members. The list is moderated by FHS and serves as a platform for the exchange of ideas. 

*Q: How was the book received?*

A: It was published by Cambridge University Press in 2012, a few months into the Arab uprisings, so there was tremendous interest. However, it quickly became clear that more should be said and we approached Richard Horton, the Editor-in-Chief of the *Lancet*, for his support with that. He agreed to publish a *Lancet* series on *Health in the Arab world* which was published in 2014. In 2016, we were chosen to lead the *Lancet*-AUB Commission on Syria, the first time that a *Lancet* commission was led by an institution from the Global South. One of the recent outputs of the commission’s work is a Global Alliance on War, Conflict and Health that was launched in May 2020. Its aim is to present the health-based argument for preventing war and armed conflict. 

Of course, we also have an impact on public health education, contributing to the strengthening of the field in the region by offering scholarships to promising undergraduate students from disadvantaged communities in Lebanon and graduate students from Lebanon, the Eastern Mediterranean Region and sub-Saharan Africa. The diversity of our student body has greatly enriched our programmes. The graduate public health programme was accredited by the Council on Education for Public Health (CEPH) in 2006 and reaccredited in 2012 and 2020, remaining the only CEPH-accredited programme in the Arab world and one of only nine accredited programmes internationally.

“We need to break the cycles of building and of loss.”

*Q: To what extent has the work of FHS impacted national policy? *

A: A lot of FHS research has fed into legislative changes on many public health issues such as air quality, water quality, food safety, salt iodization and hospital accreditation. FHS was instrumental in the promulgation of a law in 2011 banning smoking in indoor public spaces and a decree in 2012 banning the worst forms of child labour. We have a good relationship with the Ministry of Public Health, our students train there and the former director-general actually taught at FHS. The FHS Knowledge to Policy Center (K2P) hosts policy dialogues and produces evidence summaries, policy briefs and user-friendly summaries for the media. It is currently doing a lot of work on COVID-19. The work of K2P, and Center for Research on Population and Health, is complemented by that of the FHS Center for Public Health Practice which promotes evidence-based practice, builds capacity, and facilitates exchange between FHS faculty and students and the community. So, there is a lot of policy-related activity at FHS and it does bring about change. Is it enough? No. The needs are tremendous and there are frustrations regarding the government’s implementation of policy in certain areas. Needless to say these frustrations are being exacerbated in the political and financial crisis that followed last year’s explosion. 

*Q: Were you in Beirut when the explosion happened?*

A: Yes, I was at my home. We only lost one window, but it felt like the world had come to an end. That noise and the shock of the blast wave going through. The impact of the explosion is still being felt of course. It was not just the people who died or were injured, or the thousands made homeless or the billions in property destroyed, it was the feeling that our dignity as human beings had been taken away. That ‘they’ – by which I mean the political leaders who allowed that terrible thing to happen – took everything from us. So now we are fighting back. 

*Q: What are you doing?*

A: We started an initiative called Khaddit Beirut (*Khaddit* means Shake Up) that wants to rebuild better and fairer. We are now more than 100 professionals and practitioners, working on four main initiatives: environmental health (which I am leading), community health, education, and small businesses. One project specifically, under the environmental health initiative, has created a consortium between researchers and practitioners at the AUB, the Lebanese University, the University of Saint Joseph, WHO, and institutes in France and the United States. My institution-building skills developed over 12 years of being dean have come in handy. 

*Q: And the resilience developed over so many years of adversity? *

A: I have an issue with the term resilience. You do what you have to do. But you feel like Sisyphus. You build over decades and then *woosh!* – a war, a crisis, an explosion and everything is swept away. But here I am, here we are, trying to help rebuild our country again. It can be discouraging sometimes. But they cannot take away everything. Some things remain. The human capital, the knowledge, the experience, the passion. This is the challenge of working in a context of uncertainty. The region is in turmoil and hosts the majority of refugee and displaced populations globally. Lebanon, in addition, is facing a financial crisis. The Lebanese pound, in purchasing parity terms, is worth one eighth of what it was two years ago. The Lebanese are suffering. Many are leaving. So it is not enough to rebuild. We need to build better, build fairer. Not just in Beirut or Lebanon but in the region and globally. Together, we should continue to fight for an equitable world where people live in dignity. A world with no conflicts or violence, built on peace, built on justice. We need to break the cycles of building and of loss. 

